# Nanomechanical Induction of Autophagy‐Related Fluorescence in Single Cells with Atomic Force Microscopy

**DOI:** 10.1002/advs.202102989

**Published:** 2021-10-28

**Authors:** Bin Li, Yuhui Wei, Qian Li, Nan Chen, Jiang Li, Lin Liu, Jinjin Zhang, Ying Wang, Yanhong Sun, Jiye Shi, Lihua Wang, Zhifeng Shao, Jun Hu, Chunhai Fan

**Affiliations:** ^1^ CAS Key Laboratory of Interfacial Physics and Technology Shanghai Institute of Applied Physics Chinese Academy of Sciences Shanghai 201800 China; ^2^ Shanghai Synchrotron Radiation Facility Zhanjiang Laboratory Shanghai Advanced Research Institute Chinese Academy of Sciences Shanghai 201210 China; ^3^ University of Chinese Academy of Sciences Beijing 100049 China; ^4^ School of Chemistry and Chemical Engineering Frontiers Science Center for Transformative Molecules and National Center for Translational Medicine Shanghai Jiao Tong University Shanghai 200240 China; ^5^ State Key Laboratory for Oncogenes and Bio‐ID Center School of Biomedical Engineering Shanghai Jiao Tong University Shanghai 200240 China

**Keywords:** atomic force microscopy, autophagy, fluorescence, intercellular transmission, nanoacupuncture

## Abstract

Mechanistic understanding of how living systems sense, transduce, and respond to mechanical cues has important implications in development, physiology, and therapy. Here, the authors use an integrated atomic force microscope (AFM) and brightfield/epifluorescent microscope platform to precisely simulate living single cells or groups of cells under physiological conditions, in real time, concomitantly measuring the single‐cell autophagic response and its transmission to neighboring cells. Dual‐color fluorescence monitoring of the cellular autophagic response reveals the dynamics of autophagosome formation, degradation, and induction in neighboring contacting and noncontacting cells. Autophagosome formation is dependent on both the applied force and contact area of the AFM tip. More importantly, the enhancement of the autophagic responses in neighboring cells via a gap junction‐dependent mechanism is observed. This AFM‐based nanoacupuncture platform can serve as a tool for elucidating the primary mechanism underlying mechanical stimulation of living systems and other biomechanical therapeutics.

## Graphical abstract

An integrated atomic force microscope and brightfield/epifluorescent microscope platform is employed to quantify real‐time force application at the single cell level, which reveals the dynamics of autophagosome formation, degradation, and induction in neighboring contacting and noncontacting cells.

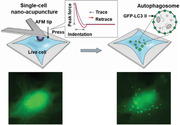

## 1 Introduction

Biomechanical stimulation is known to play key roles in biological processes, such as cell growth, differentiation, and communication.^[^ ^1^ ^]^ Recent studies have confirmed that mechanical stimulation can induce autophagy,^[^ ^2^ ^]^ which plays a housekeeping role for cells to maintain intracellular homeostasis and prevent diseases, such as cancer, neurodegeneration, cardiomyopathy, diabetes, liver disease, autoimmune diseases, and infections.^[^ ^3^ ^]^ Therefore, it is of great importance to well understand the correlation between mechanical stimulation on cells and cellular autophagy response. Being able to describe a relationship of nanoscale mechanical force on the response of autophagy at the single‐cell level is of particular interest, because it will shed light on the quantitative data that would be hidden in measurements involving a large number of cells. However, there is a lack of investigation platform that is capable of both precise generation and modulation of mechanical forces and real‐time monitoring of autophagy.

Atomic force microscopy (AFM) provides an ideal actuator and recorder of mechanical forces on cells,^[^ ^4^ ^]^ in terms of spatial precision (nanometers) and force sensitivity (piconewtons). Under the premise that compressive force is the driving stimulus to account for mechanical autophagic response we have developed a two‐component system to determine the biological effects of nanoscale compression. The system is composed of: 1) a precise and nondestructive spatiotemporal compressive force applicator based on AFM; and 2) a fluorescent optical readout of cellular responses. By applying such defined force to cells and recording the generation of autophagosomes via the fluorescent cell reporter, we can use the dual modality epifluorescent/AFM to investigate in real time the cellular response. We thus aim to quantify force application at the single cell level (dubbed nanoacupuncture) using AFM.

Traditional acupuncture may be one of the best model systems to study the correlation between mechanical force and autophagy, since at the most basic level, acupuncture is the application of mechanical forces or electrical stimulation to cells. The most commonly practiced acupuncture uses mechanical stimulation, which requires needle insertion/penetration of the skin. The compressing force on the cells exerted by the needle is the most critical because a common procedure is performed for all the acupuncture treatments during which needle compresses cells. Although acupuncture‐induced autophagy is a common response at the animal level, it is not clear whether it is a direct or indirect consequence of local mechanical stimulation at the single cell level. To quantitate the compressive force aspects of acupuncture, more accurate information about the mechanical force necessary to induce autophagy is important.

In this study, we focused on autophagy as a cellular response to understand the underlying mechanism of acupuncture at the single‐cell level in a quantitative manner under physiological conditions. Using an AFM integrated with a fluorescence microscope, we modeled acupuncture conditions on single cells by applying well‐defined compressive forces to a cell for defined time periods using AFM probes of known shape and size, a process we have termed nanoacupuncture. The autophagic response was determined by recording the optical output of the fluorescent reporter of cellular autophagy, that is, GFP‐fused LC3 protein (GFP‐LC3). The AFM tip serves as an actuator and detector of mechanical forces on cells,^[^ ^4^ ^]^ with excellent spatial precision (nanometers‐nm) and force sensitivity (piconewtons‐pN). Through the application of defined mechanical forces and recording the autophagosomes via the fluorescent reporters, we have examined the relationship between mechanical stimulation and autophagy to understand the cellular response in a quantitative manner. In this study, we selected three different tissue culture cell lines, that is, HeLa (an epithelial cell line), N2A (a neural cell line), and PC12 (induced to neuronal phenotype), as test models. This integrated approach provides a new single‐cell platform to study the mechanism of nanoacupuncture in a quantitative manner.

## 2 Results

### 2.1 Nanoacupuncture on Single Cells

To establish and refine the experimental setup of nanoacupuncture at the single‐cell level, AFM tips were modeled as acupuncture needles. Nanomechanical stimulation was carried out using AFM tips of varying tip diameters (20 nm–2.5 µm) and shapes (conical to spherical) (Figure S1a,b, Supporting Information). The tips of standard acupuncture needles can be as small as 2.0 µm, comparable to the 2.5 µm diameter AFM tips used in this study (Figure S1c, Supporting Information). To prevent damage to the cell membrane, AFM cantilevers with low spring constants (<0.35 N m^−1^) were used for force indentation as well as for imaging.

To exclude the possibility that the observed relationship between mechanical stimulation and autophagy is limited to specific cell line, three cell lines, that is, HeLa, N2A, and PC12, were selected to conduct investigation. HeLa (an epithelial cell line) cells stably expressing GFP‐LC3 and mCherry‐GFP‐LC3 have long been utilized for real‐time observation of autophagy.^[^ ^5^ ^]^ It is known that neuronal system and neuronal cells play critical roles in sensing of mechanical forces and subsequent signal transduction; moreover, dysregulation of autophagy has been demonstrated to involve in neurologic diseases. Therefore, N2A (a neural cell line) and PC12 (induced to neuronal phenotype) cells were selected to evaluate their response to mechanical stimulation and compared that to epithelial cells.

To monitor autophagy induced by nanoacupuncture, we utilized GFP‐LC3, a well‐characterized fluorescent reporter of autophagy.^[^ ^6^ ^]^ The number of fluorescent puncta (autophagosomes) correlates well with the level of autophagy.^[^ ^7^ ^]^ To confirm this basic assumption, we determined the increase in the number of fluorescent puncta with interventions previously identified as causing increased cell autophagy in HeLa cells, that is, either chloroquine or serum starvation treatments (Figure S4a–c, Supporting Information). Similarly, AFM‐based nanoacupuncture increased the number of fluorescent puncta in HeLa cells (Figures S4d–h and S5, Supporting Information).

### 2.2 Quantifying Nanoacupuncture‐Induced Autophagy

**Figure 1:**
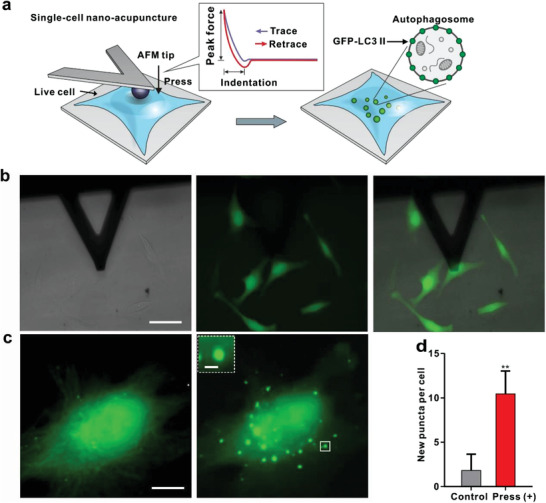
Autophagosome formation induced by AFM nanoacupuncture at the single‐cell level. a) Schematic illustration of AFM nanoacupuncture: a compressive force was applied to the plasma membrane of HeLa cells stably expressing GFP‐LC3 using AFM tip. The force–distance curve of the AFM tip approaching and retracting from the cell surface is used for the determination of indentation under a defined loading force. The nanoacupuncture‐induced autophagosome formation, which is illustrated by the accumulation of GFP‐LC3 fluorescent puncta and detected by the combined epifluorescence/AFM microscope. b) DIC image of the AFM tip (left), fluorescence image of the same view area (middle), and the merged image (right), Scale bar = 50 µm. c) Fluorescence image of a single cell before and after a 45‐min nanoacupuncture, Scale bar = 5 µm. Inset: enlargement of a single GFP‐LC3 fluorescent puncta (Scale bar = 0.5 µm). d) Summary of the number of intracellular puncta before (control) and after nanoacupuncture (press [+], *n* = 12; data were presented as mean ± SD). Loading force = 5 nN, time = 45 min, and spherical AFM tip diameter = 2.5 µm. **p < 0.01, Student's *t*‐test.

To quantify autophagy in response to nanoacupuncture in real time, we employed a temperature‐controlled, integrated imaging setup comprised of both AFM and fluorescence microscopes (**Figure** **1**a–d and Figures S2 and S3, Supporting Information). The spatial location, area of stimulation, and applied force on the cells were controlled by adjusting the settings of the AFM and tip parameters. Cellular response to a given stimulus was characterized by i) the number of autophagosomes formed per cell and ii) the percentage of cells showing such response. The autophagic response was induced by the AFM using two approaches: i) as a function of the applied force for a constant tip size, and ii) constant force with varying AFM tip size. The former reveals the relationship of indentation depth to the applied force, and the latter, the interaction area of the applied force. These parameters quantitatively characterize the autophagic response to defined mechanical stress via nanoacupuncture.

**Figure 2:**
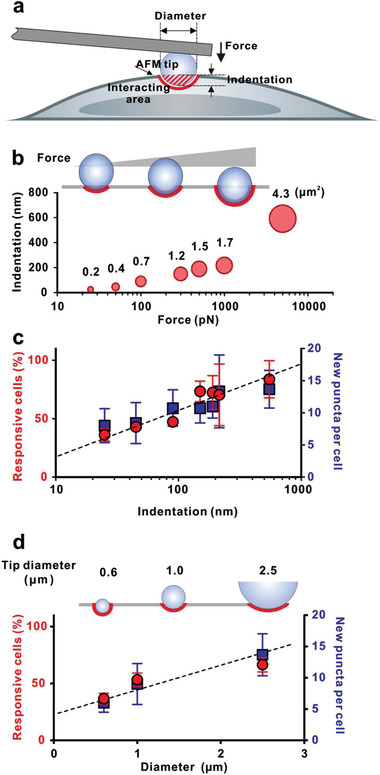
Quantitative analysis of nanoacupuncture‐induced autophagosomes in HeLa cells stably expressing GFP‐LC3. a) Schematic illustration and b) the experimental data of the correlation between the loading force, contact area, and indentation on a cell membrane using a 2.5 µm diameter spherical‐shaped AFM tip (*n* = 6; data were presented as mean ± SD). c) Percentage of cells showing autophagosomes in response to nanoacupuncture, and the corresponding number of newly formed GFP‐LC3 puncta in each cell as a function of indentation. Loading force = 25, 50, 100, 300, 500, 1000, and 5000 pN (*n* = 12, 11, 9, 10, 7, 10, and 8, respectively; data were presented as mean ± SD). Spherical‐shaped AFM tip diameter = 2.5 µm, and nanoacupuncture time = 45 min. d) Correlation of autophagosome response and diameters of spherical‐shaped AFM tip. The diameter of spherical‐shaped AFM tip is ≈0.6, 1.0, and 2.5 µm (*n* = 14, 11, and 8, respectively; data were presented as mean ± SD). Loading force = 5 nN, nanoacupuncture time = 45 min.

Using a 2.5 µm diameter spherical AFM tip, a series of well‐defined forces ranging from 25 pN to 5 nN were applied to GFP‐LC3 expressing HeLa cells for a 45‐min nanoacupuncture (**Figure** **2**a). The cellular deformation increased with the applied force. For example, a 25 pN force induced an elastic deformation of ≈25 nm in the cell membrane, which increased to ≈150 nm for 300 pN and further increased to ≈190–600 nm for a force of 500–5000 pN (Figure 2b and Table S1, Supporting Information). Throughout the experimental protocol, the cells retained their morphological integrity as ascertained by the light microscopy (Figure 1b,c), and we did not observe significant change of Young's modulus throughout the experimental protocol (Figure S6a,b, Supporting Information), which suggested the cellular membrane remained intact and no mechanical defects were produced during nanoacupuncture process. Recorded relaxation and force–distance (*F*–*D*) curves (Figure S3c,d, Supporting Information) represented possible responses of cellular rearrangements.

The degree of autophagic response was force dependent, that is, indentation dependent (Figure 2c and Table S1, Supporting Information). A small force of ≈25 pN induced a response in ≈35% of HeLa cells; the percentage of responsive cells increased to ≈75% for an applied force of 300 pN and the average number of newly formed puncta (autophagosomes) in each cell increased threefold (Figure 2c). For forces <300 pN, the response percentage and the number of puncta per cell increased continuously with increasing force. For forces between 300 and 1000 pN, the autophagic response plateaued and remained stable, with ≈75% of cells showing an autophagic response. For 5 nN applied force, ≈85% of cells displayed an autophagic response. We found a similar response in epithelial (HeLa) and neuronal (N2A and PC12) cells (Figure S7, Supporting Information). Although the response percentage of PC12 was significantly lower than that of HeLa and N2A, they showed no apparent changes in cellular morphology after applying a compressive force of 5 nN (Figures S8–S12, Supporting Information). This indicates that force‐induced autophagy is possibly a general phenomenon among different cell types.

We examined the autophagic response as a function of AFM tip diameter with a constant applied force. We applied 5 nN force for 45 min, using AFM tips of diameters of 0.6, 1.0, and 2.5 µm (Figure S13, Supporting Information). The approximate tip‐cell membrane interaction areas for these tips are ≈0.5, 1.5, and 4.6 µm^2^, respectively. Cell indentation depths for these three tips, derived from the *F*–*D* curves, are comparable to each other (Figure S13, Supporting Information). After 45 min nanoacupuncture at 5 nN, the level of autophagic response is well correlated with the diameter of AFM tips (Figure 2d and Table S1, Supporting Information). The response percentage and the average response level in each cell increased with the tip diameter. These results suggest that for the same AFM force, the level of autophagic response is dependent on the interacting area between the AFM tip and cell membrane.

### 2.3 Dynamics of the Autophagosome Formation

The dynamics of autophagosome formation was examined by time‐lapse imaging during the 45 min mechanical stimulation with 5 nN force with a 2.5 µm diameter tip. The number of GFP puncta increased over time in HeLa cells stably expressing GFP‐LC3 (Figure S14, Supporting Information). The increase in puncta reached saturation levels ≈30 min after beginning nanoacupuncture. The average number of newly formed puncta in each responsive cell increased by more than fivefold compared to unstimulated cells.

**Figure 3:**
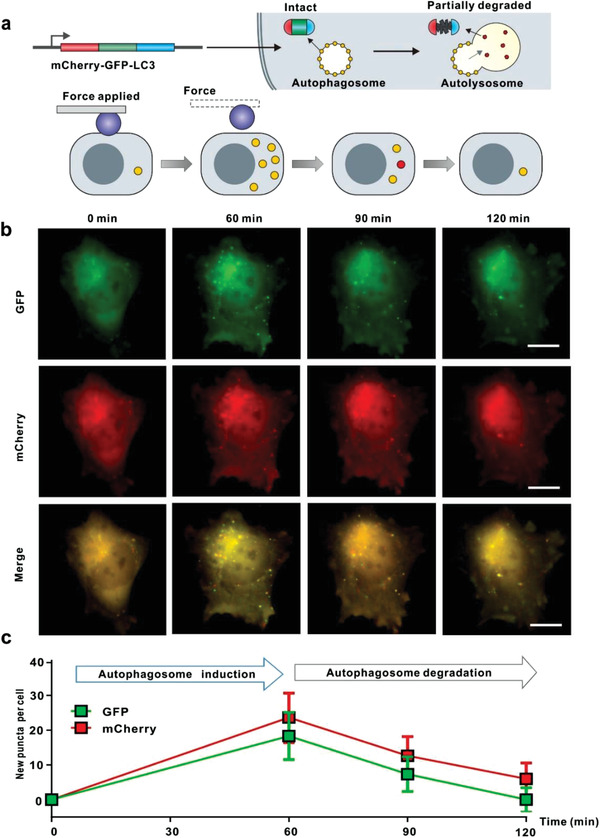
Dynamics of nanoacupuncture‐induced autophagosome formation. a) Schematic illustration of the change of observed LC3 puncta fluorescence at times indicated. b) Fluorescence images of a single HeLa cell transiently expressing cherry‐GFP‐LC3. Loading force = 5 nN. Spherical‐shaped AFM tip diameter = 2.5 µm, and nanoacupuncture time = 60 min. c) Average number of new puncta in each cell at different time points (*n* = 3; data were presented as mean ± SD), Scale bar = 10 µm.

Autophagosome formation and degradation rates determine their number within the cell. To examine the relative role of these processes, we fused the red fluorescent protein (mCherry) to GFP‐LC3 to make a dual‐labeled LC3 autophagosome reporter (mCherry‐GFP‐LC3). The relative abundance of GFP and mCherry populations enables us to differentiate the newly formed population from autophagosomes undergoing degradation through the lysosomal pathway. The green fluorescence of GFP will decrease as it enters the acidic lysosome since it is pH‐sensitive, while the red fluorescence of mCherry will be retained as it is pH insensitive (**Figure** **3**a).^[^ ^8^ ^]^ As expected, 30 min after nanoacupuncture (5 nN, 2.5 µm diameter tip), the average number of GFP puncta in responsive cells was ≈18 compared to ≈24 of mCherry puncta (Figure 3b,c). 30 min after the removal of mechanical stimulus, the total number of fluorescent puncta decreased, and the number of GFP puncta (≈7) to mCherry (≈12) decreased, suggesting that the autophagosomes are degraded in lysosomes, and the autophagy response was abrogated.

### 2.4 Nanoacupuncture Affects the Autophagic Response of Adjoined Cells via Cell–Cell Communication

**Figure 4:**
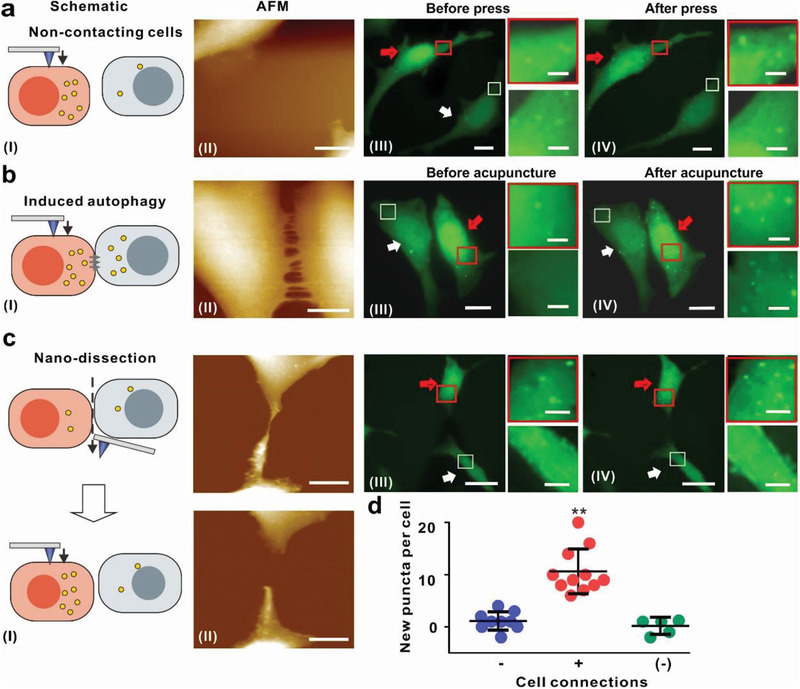
Nanoacupuncture‐induced autophagosome formation in adjacent contacting HeLa‐GFP‐LC3 cells. Nanoacupuncture‐induced autophagy a) in a cell pair without connection, b) in a cell pair with direct connection, and c) in a cell pair separated by nano‐dissection. I) the schematic of the cell pair; II) AFM image (height mode, Scale bar = 10 µm); III) the GFP‐LC3 fluorescence image of the same cell pair before; and IV) after nanoacupuncture (Scale bar = 10 and 2 µm). The cell undergoing nanoacupuncture is indicated by red arrows and the unstimulated cell is indicated by white arrows. d) Average number of new puncta in each single unstimulated cell. Cell pairs without connections (−, *n* = 8; data were presented as mean ± SD); cell pairs with connections (+, *n* = 11; data were presented as mean ± SD); cell pairs with connections resected ([−], *n* = 5; data were presented as mean ± SD). Loading force = 5 nN; cone‐shaped AFM tip diameter = 20 nm; nanoacupuncture time = 45 min. *p < 0.05, **p < 0.01, Student's *t*‐test.

To understand whether nanoacupuncture could affect the physiology of adjoining contacting cells, we examined the autophagic response of cells surrounding the stimulated cell (**Figure** **4** and Figures S15 and S16, Supporting Information). Cells stimulated directly (5 nN, cone‐shaped 20 nm tip) for 45 min generated new puncta (cells indicated by red arrows in Figure 4 and Figure S15, Supporting Information). Increased puncta were also observed in cells contacting the stimulated cells, that is, those cells not directly stimulated with the AFM tip (cells indicated by the white arrow in Figure 4b and Figure S15a–d, Supporting Information). When autophagosome formation occurred in the stimulated cell, ≈80% of the contacting cells showed increased puncta (Figure S15f, Supporting Information). This response is comparable to the case of direct stimulation, where ≈85% of cells (at 5 nN, 45 min) recorded autophagosome production (Figure S15e, Supporting Information). This transmitted nanoacupuncture‐induced autophagy phenomenon was observed in both the contacting monocultures of HeLa cells and N2A cells (Figure S15a,c,g, Supporting Information) as well as in cocultures of HeLa and N2A cells (Figure S15b,d,g, Supporting Information).

**Figure 5:**
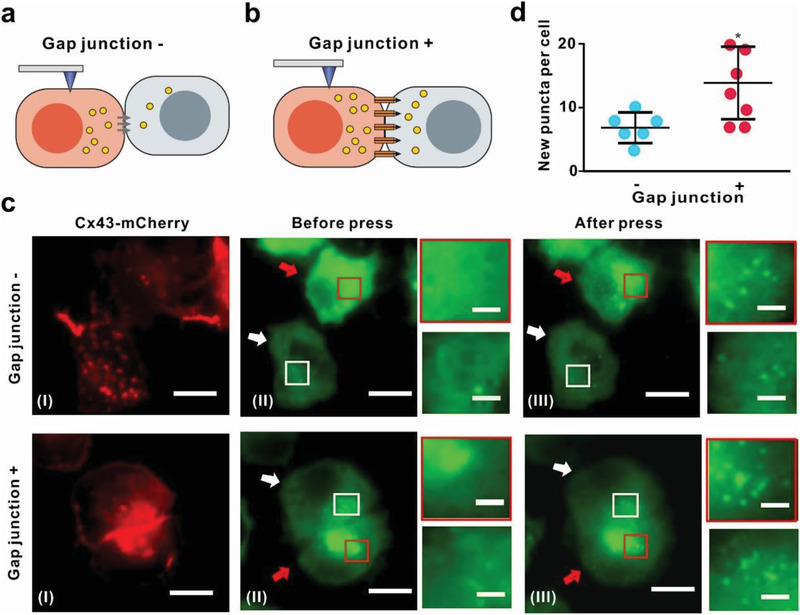
Gap junction contributed to nanoacupuncture‐mediated autophagosome formation in adjacent contacting N2A‐GFP‐LC3 cells. Schematic illustration of nanoacupuncture‐induced autophagy in a single connected N2A‐GFP‐LC3 cell pair a) without and b) with gap junction. c) Fluorescence images of a single connected N2A‐GFP‐LC3 cell pair. (I) Cx43‐mCherry fluorescence representing the gap junction plaques, and (II) GFP‐LC3 fluorescence indicating autophagosomes formation before and (III) after nanoacupuncture (scale bar 10 µm and 2 µm). The cell undergoing nanoacupuncture is indicated by red arrows and the unstimulated cell is indicated by white arrows. d) Average number of new puncta in each single unstimulated cell. Cell pairs without gap junction (−, *n* = 6; data were presented as mean ± SD); cell pairs with gap junction (+, *n* = 7; data were presented as mean ± SD). Loading force = 5 nN; cone‐shaped AFM tip diameter = 20 nm; nanoacupuncture time 75 min. *p < 0.05, **p < 0.01, Student's *t*‐test.

We explored the mechanism by which the induction of autophagosomes was transmitted. Except cells without connection, we observed contact lengths between cells extending more than 2 µm (Figure 4 and Figure S16b, Supporting Information). To investigate whether such contacts play a role in the transmission, we resected the connection between paired HeLa‐GFP‐LC3 cells using the AFM tip as a nano‐dissector (**Figure** **5**) and repeated the experiments. The resected noncontacting unstimulated cells failed to show an autophagic response, that is, no new puncta were observed (Figure 5, white arrow cells). This suggests that cell–cell contact is vital for the transmission of the autophagic response. Similar results were observed for N2A‐GFP‐LC3 cells (Figure S16, Supporting Information). Autophagosome was induced in nonstimulated contacting cells, but separated cells failed to show an increase in new puncta. We then examined whether the autophagosomes could be transported directly between contacting cells. We cocultured stably transfected HeLa‐GFP‐LC3 cells (green fluorescence) with unlabeled HeLa cells to enable direct contact. We selected contacting pairs of cells and subjected the HeLa‐GFP‐LC3 expressing cells to nanoacupuncture (5 nN, 45 min, 20 nm tip) (Figure S17, Supporting Information). Significant puncta formation was observed in the HeLa‐GFP‐LC3 cells that were directly stimulated (Figure S17d, Supporting Information, indicated by red arrows). However, no green puncta were observed in the contacting cells without GFP expression. In addition, green puncta were also observed in the HeLa‐GFP‐LC3 cells while the cell without GFP expression was stimulated. This suggests that autophagosomes (puncta) do not move between contacting cells. It also suggests a separate cell‐to‐cell communication mechanism that transmits signals for autophagosome formation to the neighboring (unstimulated) cells.

Gap junction signaling is the primary mechanism for direct cell‐to‐cell communication between adjacent cells.^[^ ^4^, ^9^ ^]^ The three cell types utilized in this study have low levels of gap junction intercellular communication (GJIC) through the endogenous connexin. To ascertain the role of GJIC on the transfer of autophagy signals between contacting cells, we examined cells with such low levels of endogenous connexin expression (N2A cells) and compared their responses following connexin transfection (Cx43‐mCherry, Figure 5).^[^ ^10^ ^]^ After a 75 min stimulation (5 nN, cone‐shaped 20 nm tip), the appearance of puncta was recorded in contacting but unstimulated cells with and without transfection of Cx43‐mCherry (Figure 5, cells indicated by white arrow). When Cx43‐mCherry plaques are present, the number of contacting cells responding remained the same, but a significantly higher number of puncta were observed (Figure 5, cells indicated by the red arrow, second row [GJIC +]). These results suggest that the transfer of autophagy signals from stimulated cells to nonstimulated cells occurs more readily when endogenous connexin expression is supplemented, that is, a larger amount of gap junction channels are present. This could occur either through the physical connection itself, that is, deformation of the stimulated cell is mechanically transferred across to the unstimulated cell or an autophagosome signal of less than ≈1000 Daltons.^[^ ^9c^ ^]^

## 3 Discussion

Here, we have established and applied a proof of concept of the combinatorial approach to physical and light‐based microscopy‐namely an integrated AFM/epifluorescence microscope. This combination has been applied on a multilevel systems approach. First, we have demonstrated the direct real‐time co‐imaging modalities of AFM and fluorescence microscopy, which enables individual cells identified by biological reporter expression to be characterized with the AFM. The AFM usage was demonstrated by imaging GFP‐LC3 specifically expressed in cells. We utilized the exquisite control the AFM manifests to determine multiple observational and interventional parameters: including topological determination (nm–µm scale), defined force application (pN–nN scale), defined deformation (indentation), physical measurements of compliance (Young's modulus), and direct individual cell nano‐surgery‐manipulation. The combination of the AFM with varying expression constructs including multiple fluorescent reporter constructs GFP‐LC3, mCherry‐GFP‐LC3, and Connexin 43‐mCherry demonstrates that with the appropriate excitation and emission subsystems a range of wavelengths can be imaged.

The cell population response to nanoacupuncture places into context the study of acupuncture. Acupuncture is an ancient healing method widely practiced in China and other parts of the world, including the USA.^[^ ^11^ ^]^ It is applied in the safe and effective management of a broad spectrum of ailments, including pain, Parkinson's disease, stress, asthma, and depression.^[^ ^12^ ^]^ Acupuncture relies on applying mechanical stimulation using fine‐tipped (typically <10 µm tip diameters, Figure S1, Supporting Information) needles to specific points on the body, called acupoints, located at various depths under the skin. The mechanism of action of acupuncture, however, is unclear, and there have been multiple theories put forth.^[^ ^11^, ^13^ ^]^ At its most basic, stimulation would be mechanical at the single‐cell level. During acupuncture, the cells are subjected to various types of mechanical forces, including friction, adhesion, torsion, and compression. The mechanical pressure exerted on cells appears to be the most critical factor, as most acupuncture treatments involve constant or rotating mechanical force application with micron‐sized tipped acupuncture needles for ≈30 min periods.^[^ ^11^ ^]^ Although little is known of the effects of such applied forces on individual cell responses, existing evidence suggests that mechanical stress enables induction of autophagy and one of the significant responses to acupuncture involves the induction and modulation of autophagy across cell layers.^[^ ^14^ ^]^

We reason that our approach is capable of teasing out how acupuncture can induce physiological changes in the body perhaps via epidermal or nerve cell stimulation, that is, cell population response or activation of the autonomic/peripheral nervous system.^[^ ^15^ ^]^ The cell population response to nanoacupuncture described in this work is a significant step toward a more comprehensive understanding of the cellular and molecular mechanisms underpinning the ancient art of acupuncture.

## 4 Experimental Section

### Cell Culture

HeLa, PC12, and Neuro‐2A (N2A) cells were purchased from Stem Cell Bank/Stem Cell Core Facility (SIBCB, CAS). HeLa and N2A cells were maintained in Modified Eagle's Medium (Thermo Fisher Scientific), while PC12 cells were maintained in Dulbecco's Modified Eagle's Medium (Thermo Fisher Scientific), containing 10% fetal bovine serum (Gemini, USA), 1% L‐glutamine (Thermo Fisher Scientific), and 1% penicillin/streptomycin (Thermo Fisher Scientific) at 37 °C in a humidified atmosphere containing 5% CO_2_.

### Plasmids Transfection

The following plasmids were obtained from Addgene: pBABEpuro GFP‐LC3 from Jayanta Debnath (Addgene plasmid #22405)^[^ ^16^ ^]^ and FUW mCherry‐GFP‐LC3 from Anne Brunet (Addgene plasmid #110060).^[^ ^17^ ^]^ Cx43‐mCherry was constructed on pLPCX‐Cx43‐IRES‐GFP from Trond Aasen (Addgene plasmid #65433).^[^ ^18^ ^]^ Transfection of HeLa, PC12, and N2A cells with indicated plasmids was performed with Lipofectamine 3000 (Invitrogen) according to the manufacturer's instructions. Transduced cells were grown in the medium as described above. Selection of transduced cells was performed with 1 mg mL^−1^ Geneticin (G418) (Thermo Fisher Scientific) for about 15 days. High‐expressing cell clones were identified using fluorescence microscopy. Following selection, stably transfected HeLa and PC12 cells were cultured and supplemented with 500 µg mL^−1^ G418, and N2A cells were cultured with 800 µg mL^−1^ G418.

### Atomic Force Microscopy and Fluorescence Microscopy

A Bioscope Resolve AFM (Bruker, USA) and fluorescence microscopy (Leica DMI 3000 B, Germany) were used in the present study. Probes with various tip diameters of 20–25000 nm were used in AFM experiments. The nominal spring constant of probes was ranged within 0.1–0.35 N m^−1^. A PeakForce loading on cell samples was within 25 pN–5 nN during 45–75 min nanoacupuncture.

### Image Analysis

The fluorescence images were analyzed using ImageJ and AFM images were analyzed with Nanoscope Analysis 2.0 (Bruker NanoScope Analysis 2.0 Santa Barbara, CA, USA).

### Scanning Electron Microscopy

Needle diameters were evaluated using a scanning electron microscope (SEM) (Zeiss, LEO 1530VP). SEM was operated at 10 kV with magnification of 50–37 000.

### Statistical Analysis

All the data were collected from at least three independent experiments, and analyzed without preprocessing. Statistical evaluation was conducted using Student's two‐tailed *t*‐test with GraphPad Prism 7.0 (*p < 0.05, **p < 0.01, ***p < 0.001). Details of data presentation, sample size (*n*), statistical analysis, and significance of differences were provided in the figure legends.

## Conflict of Interest

The authors declare no conflict of interest.

## Author Contributions

B.L., Y.W. (Wei), and Q.L. contributed equally to this work. J.H. and C.F. directed the research; B.L., J.H., Z.S., and C.F. conceived and planned the study; B.L., L.W., J.H., Z.S., and C.F. designed experiments; Y.W. (Wei) carried out AFM experiments under the supervision of B.L., Q.L., N.C., and Y.W. (Wang); L.L. performed cell culture and plasmid transfection under the supervision of N.C.; L.W. and J.L. constructed plasmids; J.Z., Y.W. (Wang), and Y.S. assisted plasmid construction and cell transfection; B.L., Y.W. (Wei), Q.L., N.C., Y.W. (Wang), L.L., J.H., and C.F. analyzed data; J.S., L.W., and Z.S. contributed to discussions and overall impact of the work; B.L., Q.L., J.H., and C.F. cowrote the paper with input from all the authors.

## Supporting information

Supporting Information

## Data Availability

Research data are not shared.
