# Supplementary material for: Nanomechanical Induction of Autophagy‐Related Fluorescence in Single Cells with Atomic Force Microscopy
Source: Adv Sci (Weinh). 2021 Oct 28;8(24):2102989. doi: 10.1002/advs.202102989 (PMC8693060; doi:10.1002/advs.202102989)
Supplement: Supplementary file 1 — Supporting Information [file ADVS-8-2102989-s001.pdf]

WILEY-VCH

© 2021 Wiley-VCH GmbH

ADVANCED SCIENCE
Open Access

Supporting Information

for *Adv. Sci.*, DOI: 10.1002/advs.202102989

Nanomechanical induction of autophagy-related fluorescence in single cells with atomic force microscopy

Bin Li[1,2†], Yuhui Wei[1,2,3†], Qian Li[4†], Nan Chen[1], Jiang Li[1,2], Lin Liu[1], Jinjin Zhang[1,2], Ying Wang[1,2], Yanhong Sun[1,2], Jiye Shi[1], Lihua Wang[1,2], Zhifeng Shao[5], Jun Hu[1,2], Chunhai Fan[4*]*

Supporting Information

Nanomechanical induction of autophagy-related fluorescence in single cells with atomic force microscopy

Bin Li[1,2†], Yuhui Wei[1,2,3†], Qian Li[4†], Nan Chen[1], Jiang Li[1,2], Lin Liu[1], Jinjin Zhang[1,2], Ying Wang[1,2], Yanhong Sun[1,2], Jiye Shi[1], Lihua Wang[1,2], Zhifeng Shao[5], Jun Hu[1,2], Chunhai Fan[4*]*

Supplementary Text

1. Experimental Section

1.1. GFP-LC3 transfection

2×10^4 HeLa and PC12 or 3×10^4 N2A cells were seeded in a standard glass-bottomed well plate (BD biosciences) and cultured in MEM (HeLa and N2A) or DMEM medium (PC12) for 24 h before treatment. Growth media were replaced before transfection with MEM or DMEM medium containing geneticin (G418) (Thermo Fisher Scientific) at a final concentration of 1 mg/mL. To each sample, 0.8 μL of Lipofectamine 3000 was added as described in the manufacturer's instructions, mixed with 300 ng GFP-LC3 plasmids (Jayanta Debnath, Addgene plasmid #22405) and 25 μL incubation reagent. Transfected cells were incubated for 3 to 5 days before clonal selection. Cells with strong fluorescence were picked under a fluorescence microscope and then digested with trypsin, diluted into single colonies suspension, and seeded onto cell culture dishes. After cell colonies with strong fluorescence were obtained, HeLa and PC12 cells were cultured in 500μg/mL G418 and N2A cells were cultured in 800 μg/mL G418, respectively.

1.2. mCherry-GFP-LC3 transfection

2×10^4 HeLa cells were seeded in a standard glass-bottomed well plates and cultured in MEM medium for 24 h before transfection. Growth media were replaced before transfection with MEM medium containing G418 at a final concentration of 1 mg/mL. To each dish, 0.8 μL of Lipofectamine 3000 was added as described in the manufacturer's instructions using Opti-MEM, mixed with 300 ng mCherry-GFP-LC3 plasmids (Anne Brunet, Addgene plasmid

#110060) and 25 μL incubation reagent. Transfected cells were incubated as described above and then cultured in MEM supplemented with 500 μg/mL G418.

1.3. Cx43-mCherry transfection

In order to study the role of gap junction communication for the transfer of an autophagic signal, we transfected endogenously low connexin expressing N2A cells with Cx43-mCherry. Cx43-mCherry plasmid was constructed from pLPCX-Cx43-IRES-GFP from TrondAasen (Addgene plasmid #65433). 3×10^4 N2A-GFP-LC3 cells were seeded in a standard glass-bottom well plate and cultured in MEM medium for 24 h. Growth media were replaced before transfection with MEM medium containing G418 at a final concentration of 1 mg/mL. To each dish, 500 ng Cx43-mCherry plasmid was added with 0.8 μl Lipofectamine 3000 and 25 μL incubation reagent according to the manufacturer's instructions. Transduced cells were grown in the medium, as described above. Adjacent contacting cells expressing Cx43-mCherry (with the formation of gap junction) were selected for nanoacupuncture experiments.

1.4. Autophagy induction

2×10^4 (HeLa and PC12) or 3×10^4 (N2A) cells were seeded in a standard confocal glass-bottom well plate (BD Biosciences) and cultured in MEM or DMEM medium (Thermo Fisher Scientific) for 24-36 h before treatment.

For nanoacupuncture experiments, 2×10^4 (HeLa and PC12) or 3×10^4 (N2A) cells were seeded in a standard confocal glass-bottom well plate (BD Biosciences) and cultured in MEM or DMEM medium (Thermo Fisher Scientific) for 24-36 h before treatment. During AFM experiments, cells were maintained in Leibovitz's L-15 medium (Thermo Fisher Scientific) in environments without CO_2 equilibration

To induce autophagy via starvation, cells were incubated in media lacking serum-opti-MEM (Thermo Fisher Scientific) for 45 min/12 h. To induce autophagy with chloroquine (CQ), cells were incubated in MEM medium containing 30 μM CQ (Thermo Fisher Scientific) for 45 min/12 h.

1.5. Cell staining

To study autophagy between N2A-GFP-LC3 and HeLa-GFP-LC3 cells, the nucleus in N2A cells were stained with Hoechst 33258 (Sigma-Aldrich, USA). 2×10^4 N2A-GFP-LC3 cells were seeded in a standard glass-bottomed well plate and cultured in MEM medium for 24 h, then incubated in MEM medium containing 5 μg/mL Hoechst 33258 for 10 min and washed for 3 times with PBS. Then to the confocal plates with Hoechst 33258 stained N2A-GFP-LC3 cells, 2×10^4 HeLa-GFP-LC3 cells were seeded, and the cell mixture was incubated in MEM medium for 12 h. The adjacent contacting cell pairs consisting of N2A-GFP-LC3 (nucleus stained with Hoechst) and HeLa-GFP-LC3 cells were selected for nanoacupuncture experiments.

1.6. Atomic force microscopy and fluorescence microscopy

A Bioscope Resolve AFM (Bruker, USA) and fluorescence microscope (Leica DMI 3000 B, Germany) were used in the present study. Fluorescence images were acquired by oil-immersion 63× (NA1.4, Leica) with 380 nm, 480 nm, and 520 nm laser lines. All living cell AFM experiments were performed in Leibovitz's L-15 cell culture medium at 37 °C and 40-50% environmental humidity.

The tip radius for the DNP-S10 cantilever was calculated by applied deconvolution on image results for the RS-12M tip-checker sample (Bruker). Other cantilevers' tip radii were calibrated by their manufacturers. Spring constants for DNP-S10 and silicon nitride probes were calibrated by the thermal noise method in Bruker Nanoscope 9.3 software.

The force on the cells was loaded in the PFQNM ramp mode. The relaxation and force-distance (F-D) curve measurements were obtained with a 4.0 μm/s ramp rate, 2.0 μm ramp size, and holding time of 2700 s or 4500 s (45 min or 75 min). The loading force applied to the samples varied between 25 pN to 5 nN, according to the different experimental protocols. For the nano-dissection, approximately 7–10 nN force was applied in PeakForce tapping mode.

Indentation depths of cells were determined from F-D curves and Young's modulus of cells was calculated using the JKR model. According to the JKR model, the applied force F and the indentation depth h are related by:[1]

$$h = \frac{a^2}{R} - \sqrt{\frac{2\pi a \Delta\gamma}{E^*}} \qquad (1)$$

$$a = \{\frac{3FR}{4E^*}\left[1 + \frac{3\pi\Delta\gamma R}{F} + \sqrt{\frac{6\pi\Delta\gamma R}{F} + \left(\frac{3\pi\Delta\gamma R}{F}\right)^2}\right]\}^{\frac{1}{2}} \qquad (2)$$

E*=E/(1-ν2) (3)

Where F is the force applied on the cell by the spherical AFM tip, Δγ is the work of adhesion, E is the elastic modulus of the cell, ν (with ν = 0.5) is the Poisson ratio of the cell, R is the tip radius, a is the contact radius, and h is the indentation depth.

For the interacting area between the cell membrane and a spherical tip, different equations were used to calculate due to different indentation depth. When indentation depth (h) is larger than the radius of the tip (R) (a), the interacting area (IA) between the cell membrane and a spherical tip is calculated following the equation $IA=2\pi R^2$. When indentation depth (h) is smaller than the radius of the tip (R) (b), the interacting area (IA) between the cell membrane and a spherical tip is calculated following the equation IA=2πRh. Schematic illustration of the correlation between the contact area, and indentation on a cell membrane using a diameter spherical-shaped AFM tip was shown as follows.

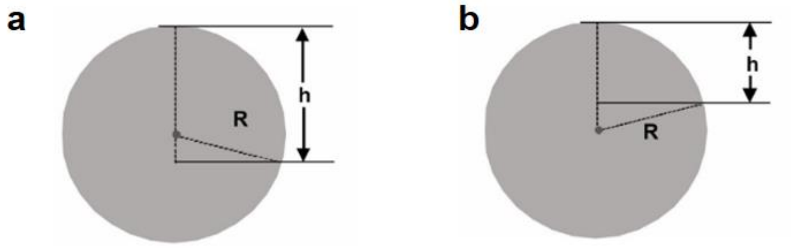

The morphological images of cells were acquired with a tapping frequency of 0.5–1.0 kHz using DNP-S10 tip (diameter of 20 nm). The PeakForce tapping amplitude was 300 nm with the setpoint 1.0 nN. Measured heights of cells before nanoacupuncture were determined from sectional profiles of three types of Cells. All AFM images and F-D curves were analyzed by NanoScope Analysis 2.0 (Bruker).

Change of occupied area of cells on Petri dishes before and after nanoacupuncture were captured using fluorescence imaging and then calculated using Image J software.

1.7 Nano-dissection of a connected cell pair

Two adjacent cells was selected with the optical microscope, and the connected area between them was scanned with AFM imaging (PeakForce tapping mode) to confirm if there was a direct physical connection between these two selected cells. To cut the connection, the cone-shaped AFM tip (20 nm diameter) was placed perpendicularly with a loading force >8 nN and a scan aspect ratio of 1/64 frame. Typically, the nano-dissection was conducted on a small scan-area (1000 nm x 15 nm) with a scanning speed of ~1 Hz, and the whole dissection process was generally longer than 5 min. After the dissection, the connected area between the two cells was scanned with AFM imaging (PeakForce tapping mode) again to confirm the success.

2. Results

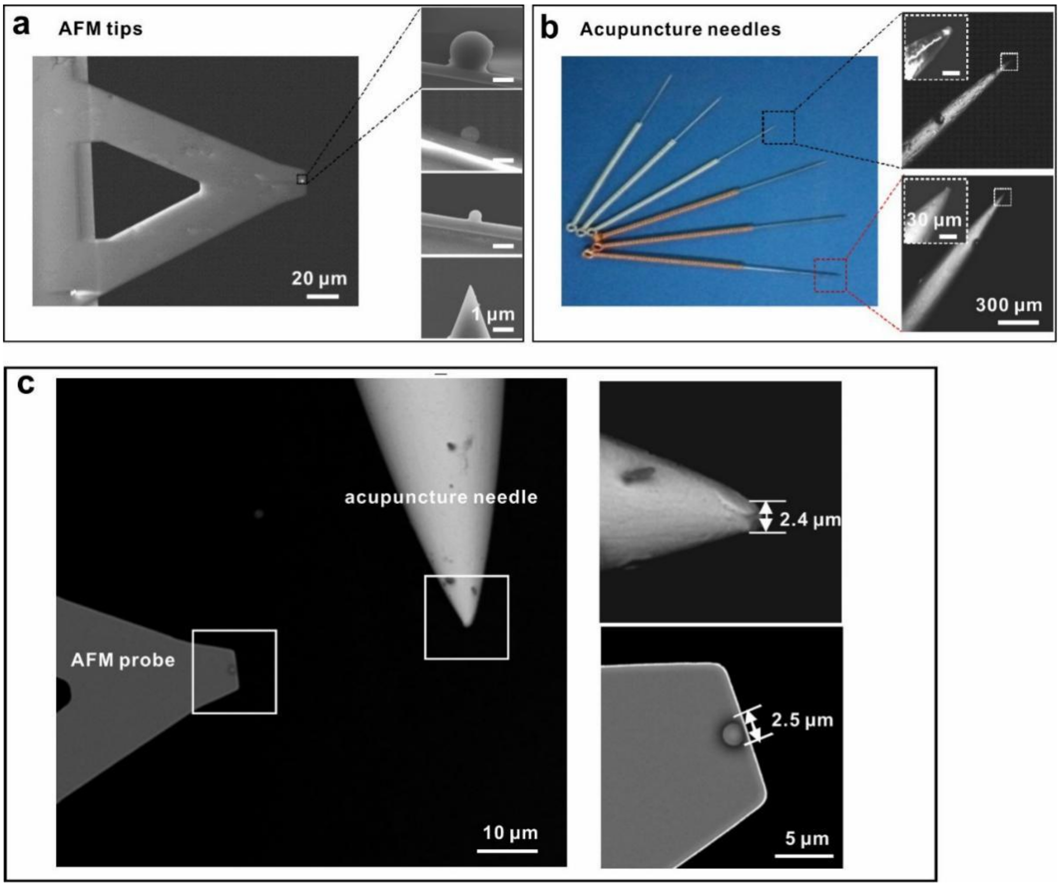

Figure S1. Size comparison of acupuncture needles and AFM probes used in nanoacupuncture. a) Scanning electron micrographs of the compliant cantilever (spring constant 0.35 N/m). Insets: SEM images of AFM tips of different diameters used for nanoacupuncture on single cells. The diameter is 2.5, 1.0, 0.6 and 0.02 µm, (from top to bottom). b) Photomicrograph of needles used in conventional acupuncture, and the corresponding SEM images of the boxed needle tip. The needle diameter of the silver (top) and copper (bottom) is <10 µm. c) SEM images of a spherical AFM probe (~2.5 µm in diameter) and a standard acupuncture needle under the same view. Left panel, the AFM probe and the acupuncture needle were placed together for simultaneous SEM imaging. Right panel, larger magnification of the white rectangles in the SEM image on the left. The tip width of the standard acupuncture needle is ~2.4 µm.

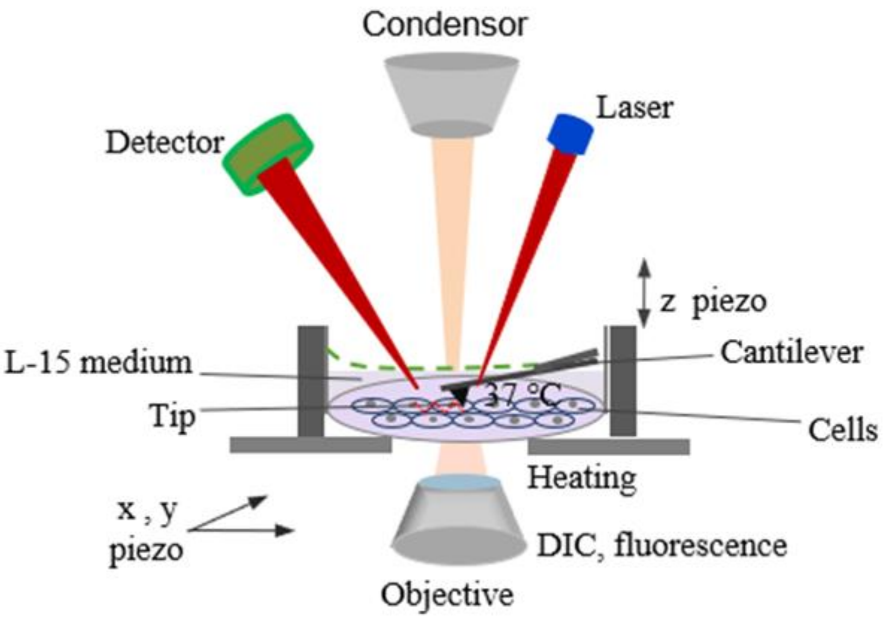

Figure S2. Schematic illustration of the integrated microscope platform.

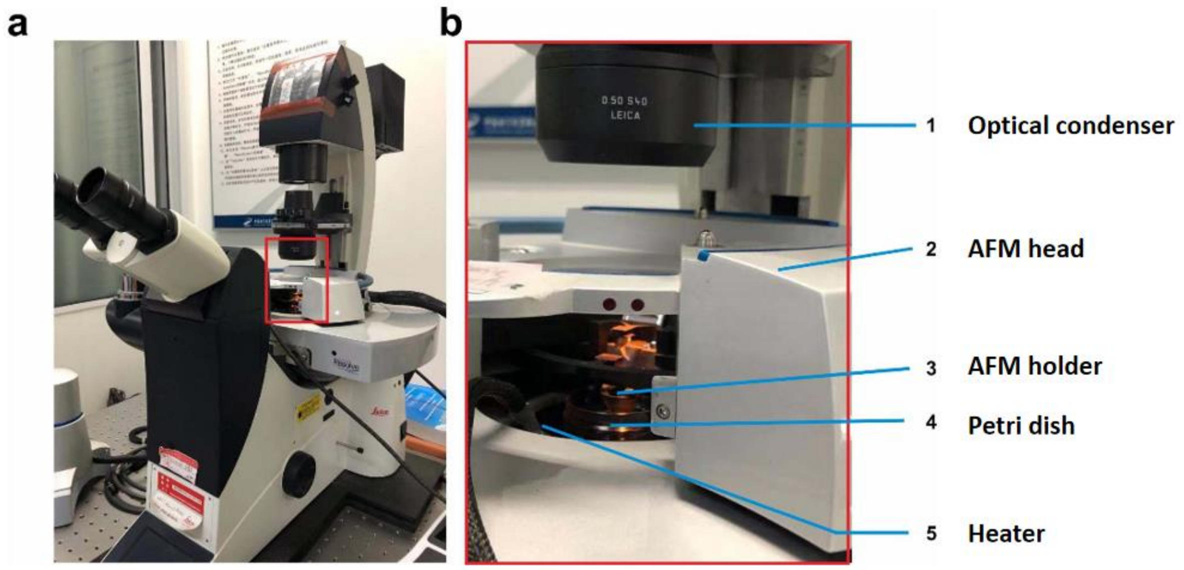

Figure S3. Photograph of the integrated microscope platform.

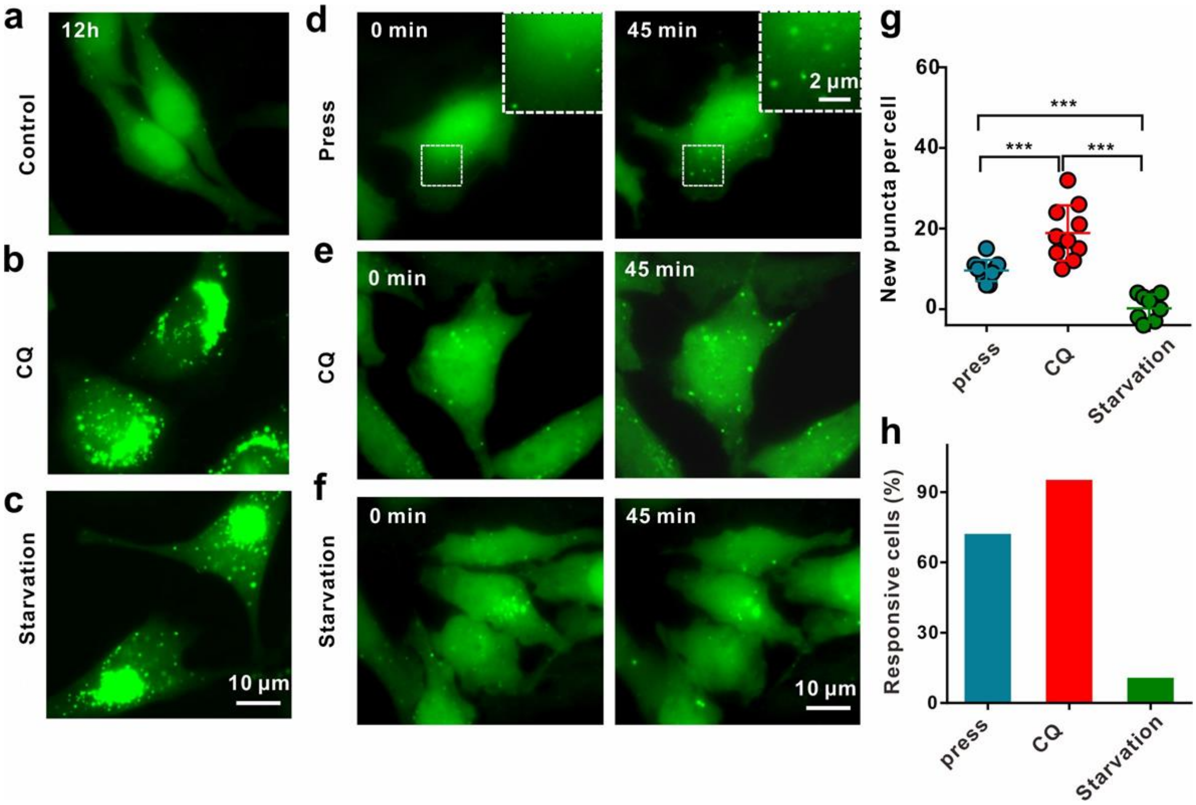

Figure S4. Formation of autophagosomes in HeLa-GFP-LC3 cells under different treatments. a) –f) Fluorescence images of HeLa-GFP-LC3 cells under different treatments. a) Blank control. b) Treatment with 30 μM CQ for 12 h. c) Serum starvation for 12 h. d) A 45-min nanoacupuncture. e) A 45-min treatment with 30 μM CQ. f) A 45-min treatment with serum starvation. g) Average number of newly formed puncta in each cell after three 45-min treatments (d-f). Nanoacupuncture: 9.6 ± 0.8, n = 10; CQ: 18.9 ± 2.2, n = 10; starvation: 0.2 ± 1.1, n = 10. Data were presented as mean ±SD. ***p<0.001, Student's t-test. h) Percentage of responsive cells after three 45-min treatments. Nanoacupuncture: 71.4%, n = 14; CQ: 94.4%, n = 36; starvation: 10%, n=40. Nanoacupuncture time, 45 min; loading force, 5 nN; sphere-shaped AFM tip diameter, 2.5 μm. ***p<0.001, Student's t-test. White scale bars represent 10 and 2 μm, respectively. The inserts are zoom-in of the white boxes.

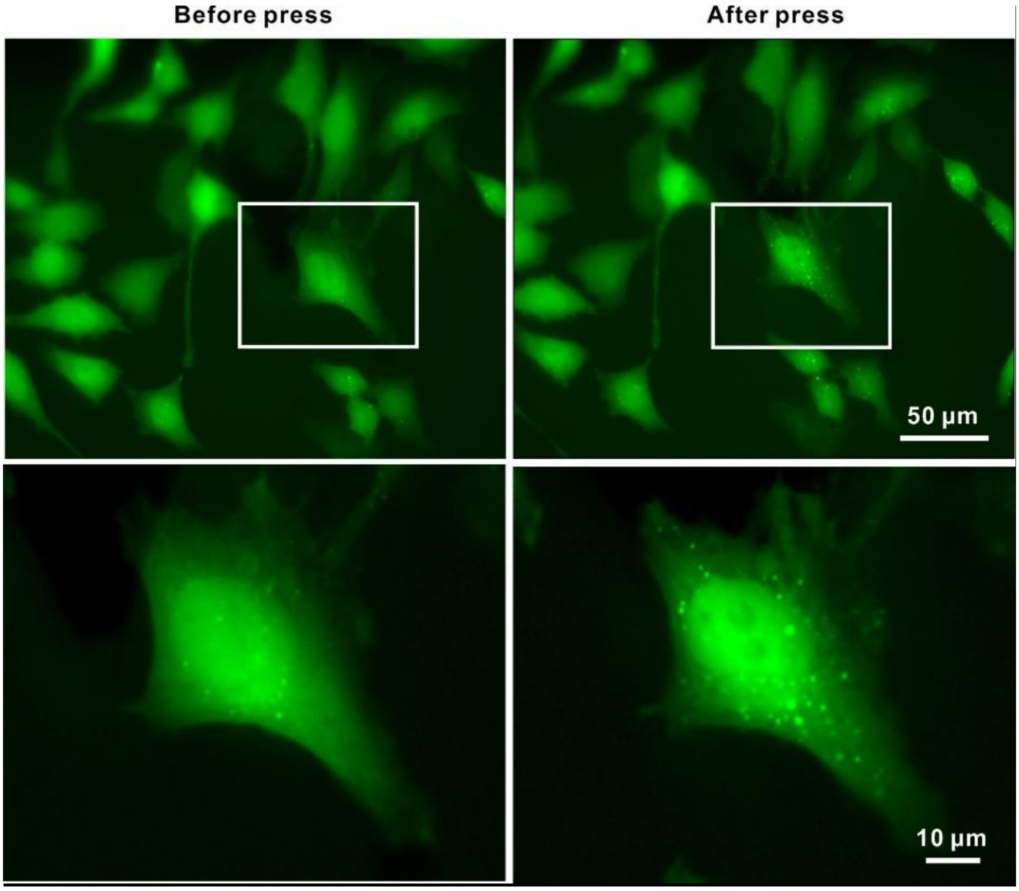

Figure S5 Fluorescence image of HeLa cells before and after a 45-min nanoacupuncture. The stimulated cell was indicated by the white rectangle. Significant damage to cell population was not observed during a 45-min nanoacupuncture process. Loading force, 5 nN; sphere-shaped AFM tip diameter, 2.5 µm.

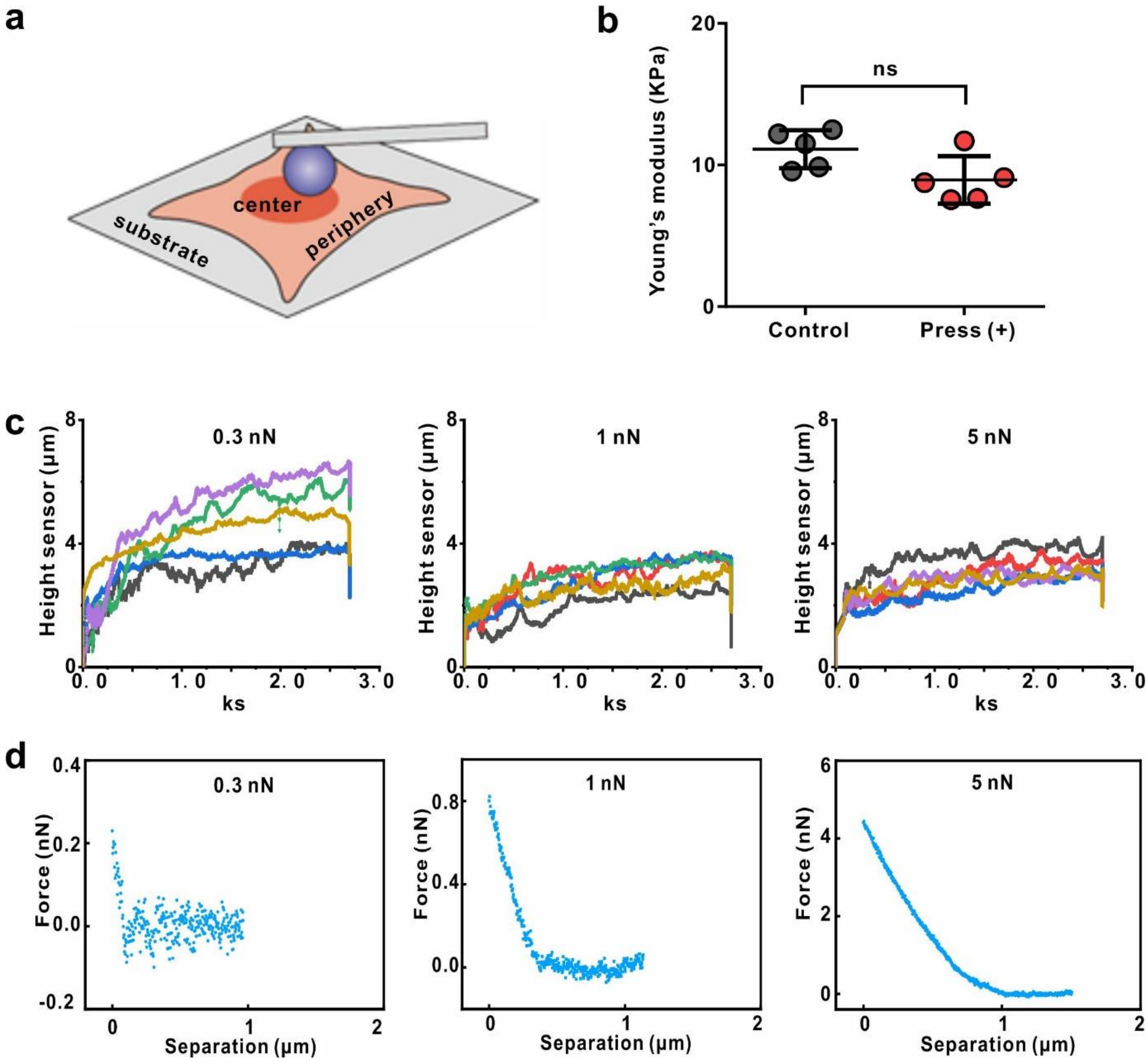

Figure S6. Response of HeLa-EGPF-LC3 cells to a constant loading force. a) Schematic representation of the spherical AFM tip pressing a single cell. b) Young's modulus of HeLa-GFP-LC3 cells before (control, n = 5) and after (press (+), n = 5) nanoacupuncture. Spherical AFM tip diameter, 2.5 μm; nanoacupuncture time: 45 min, loading force 5 nN. Data were presented as mean ±SD. c) Representative relaxation curves obtained with loading forces of 0.3, 1.0, and 5.0 nN, respectively. d) Representative F-D curves showing tip displacement ramping to the start peak point under loading forces of 0.3, 1.0, and 5.0 nN, respectively.

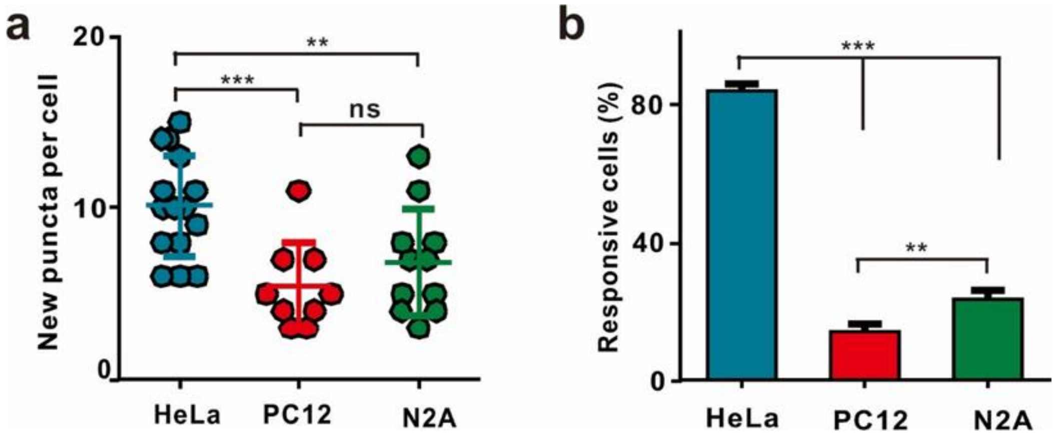

Figure S7. Nanoacupuncture induced autophagy in various cell lines. a) An average number of newly formed puncta in each responsive cell. b) Percentage of responsive cells (n=64, 71, 51). Each cell group was subjected to 5 nN nanoacupuncture with a spherical tip of 2.5 µm diameter for 30 min. Data were presented as mean ±SD. ns: no significant difference, **$p<0.01$, ***$p<0.001$, Student's t-test.

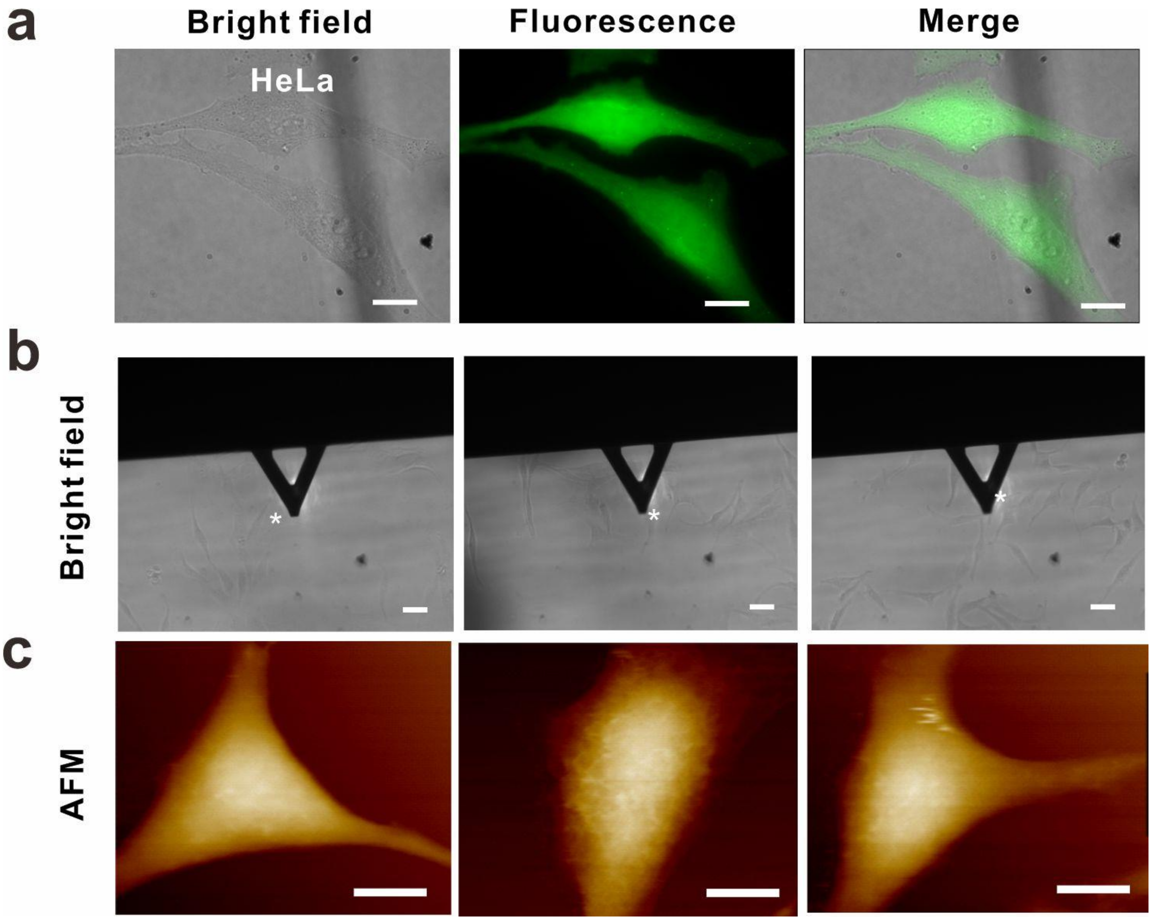

Figure S8. Geometry of HeLa cells on the integrated microscope platform. a) Representative bright field and fluorescence image of HeLa cells. b) Bright field images and c) AFM images of cells. The single cells shown in AFM images were indicated by white asterisks in brightfield images. Scale bar: 10 µm.

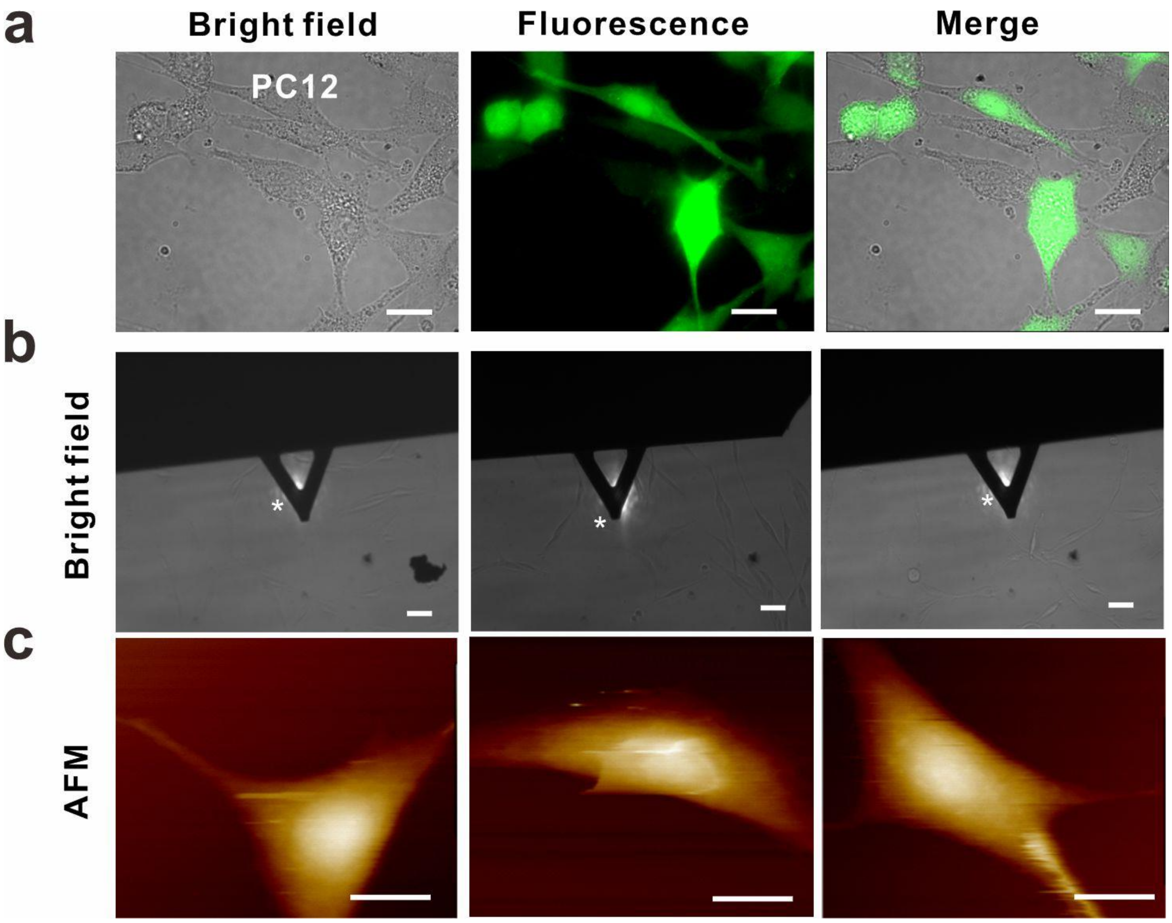

Figure S9. Geometry of PC12 cells on the integrated microscope platform. a) Representative bright field and fluorescence image of PC12 cells. b) Bright field images and c) AFM images of cells. The single cells shown in AFM images were indicated by white asterisks in brightfield images. Scale bar: 10 µm.

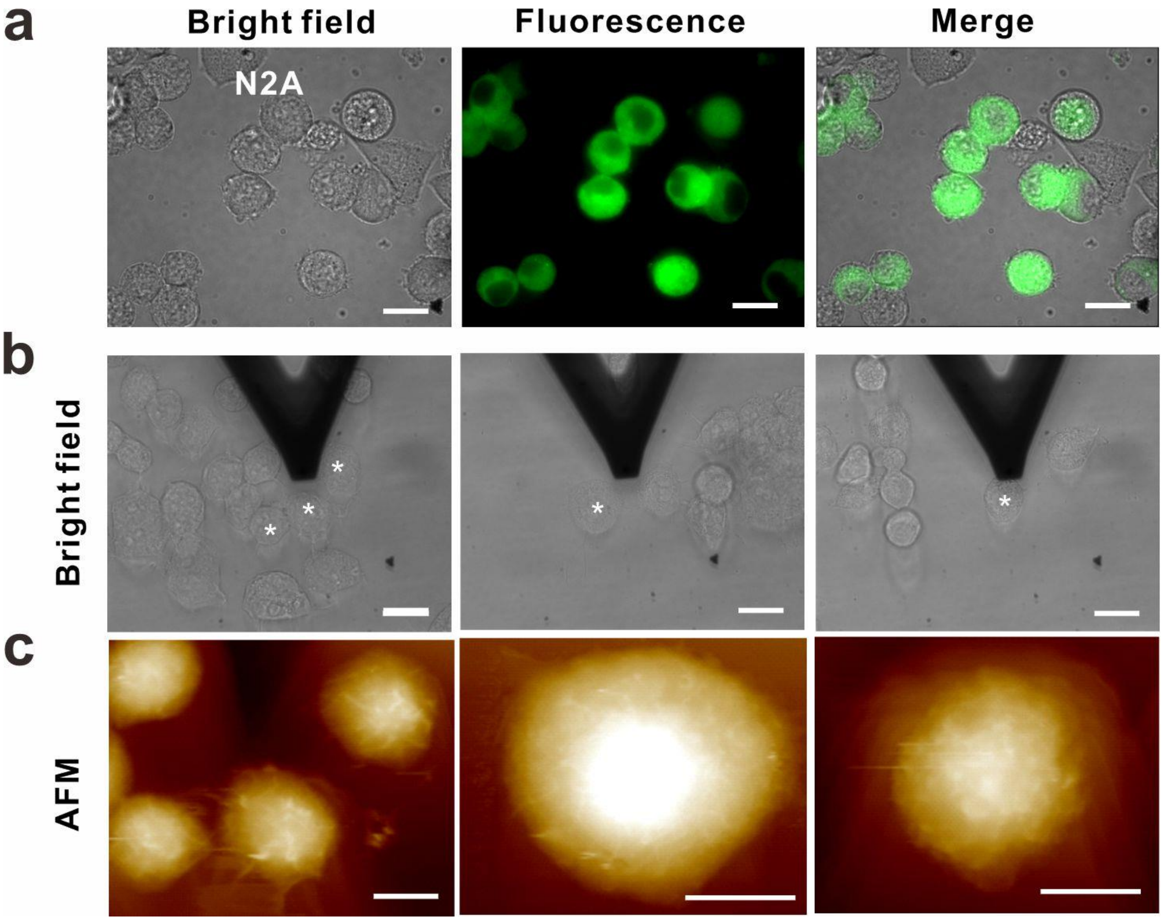

Figure S10. Geometry of N2A cells on the integrated microscope platform. a) Representative bright field and fluorescence image of N2A cells. b) Bright field images and c) AFM images of cells. The single cells shown in AFM images were indicated by white asterisks in brightfield images. Scale bar: 10 µm.

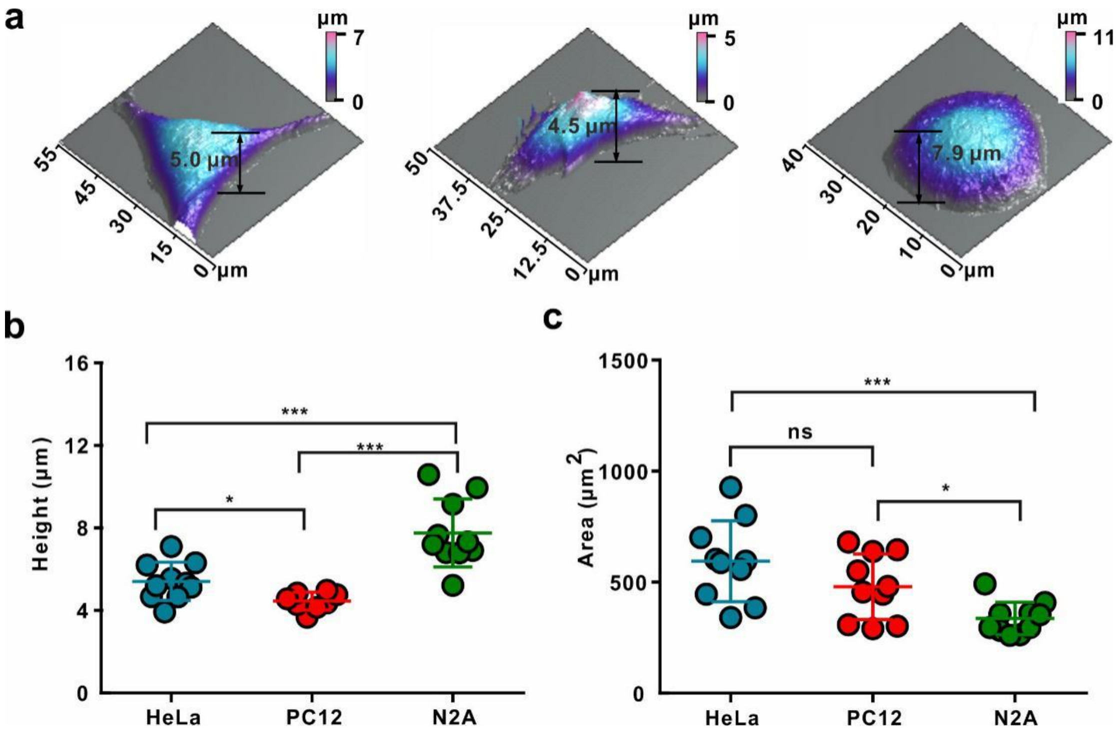

Figure S11. Geometrical features of HeLa, PC12 and N2A cells determined by AFM and Fluorescence microscopy. a) The AFM morphology image of a single HeLa (left), PC 12 (middle) and N2A cell (right) and corresponding section profiles of the three cells. b) Based on AFM sectional images, the thickness of untreated cells is determined to be 5.4 ± 0.3 µm, 4.5 ± 0.2 µm, and 7.8 ± 0.5 µm, for HeLa, PC12 and N2A cells, respectively. n = 10 for HeLa and N2A cells, n = 8 for PC12 cells. Data were presented as mean ±SD. c) Based on fluorescence images, occupied area of untreated cells on Petri dishes is determined to be 593.9 ± 57.3, 479.7 ± 46.6, and 336.8 ± 23.1 µm^2, for HeLa, PC12 and N2A cells, respectively. n = 10 cells for each group. Data were presented as mean ±SD. ns = no significant difference, *p<0.05, ***p<0.001, Student's t-test.

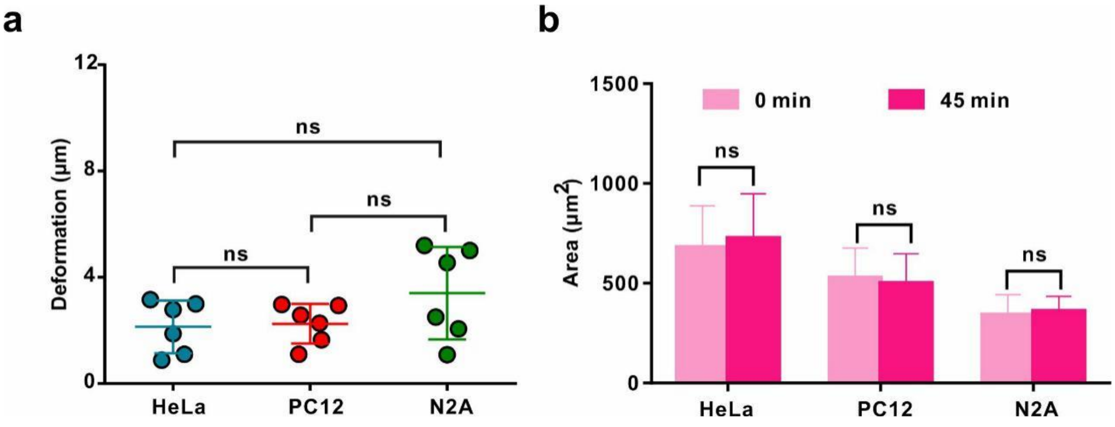

Figure S12. Change of geometrical features of HeLa, PC12 and N2A cells after nanoacupuncture. a) Mean cell indentation variation over 45 min nanoacupuncture revealed by AFM imaging results. n = 6 cells for each group. Data were presented as mean ±SD. b) The occupied area of cells on Petri dishes before and after nanoacupuncture revealed by fluorescence imaging results. n = 5 cells for each group. Data were presented as mean ±SD. Nanoacupuncture time, 45 min; loading force, 5 nN; sphere-shaped AFM tip diameter, 2.5 µm. ns = no significant difference; $^*p<0.05$, $^{***}p<0.001$; Student's t-test.

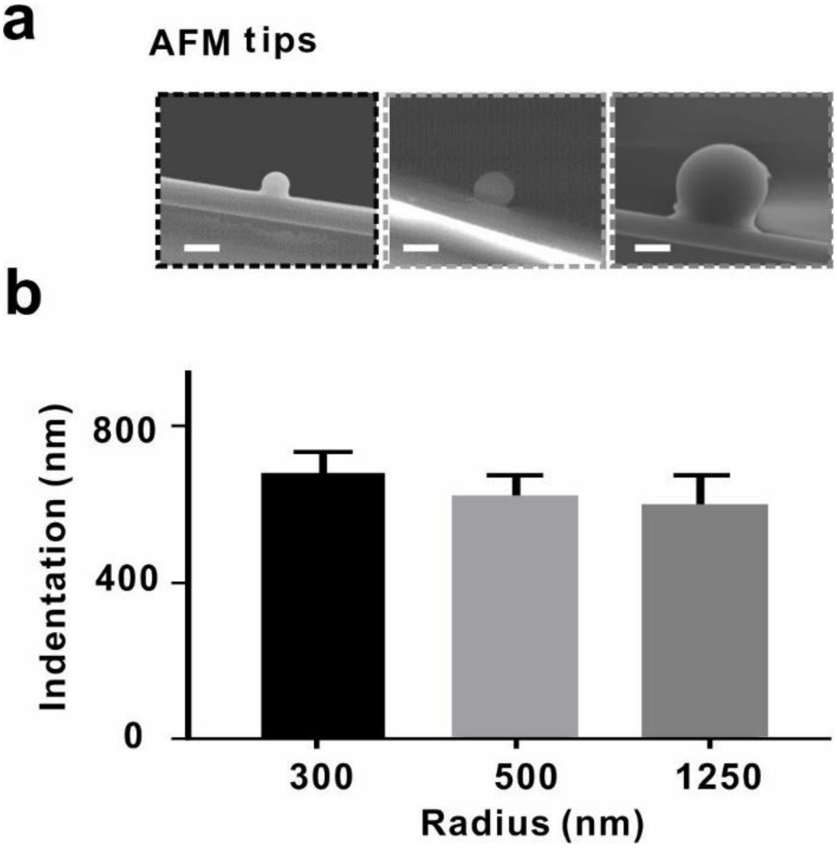

Figure S13. Different spherical AFM tips and their corresponding indentation depth on cell membrane. a) SEM images of spherical shaped AFM tip with a diameter of 600, 1000, and 2500 nm, respectively, and b) their indentation depths on cell membrane. Radius 300 nm: 672.8±21.83 nm, n=8; radius 500 nm: 610.2±24.44 nm, n=7; radius 1250nm: 591±26.38 nm, n=10. Data were presented as mean ±SD Scale bar: 1 μm; loading force: 5 nN.

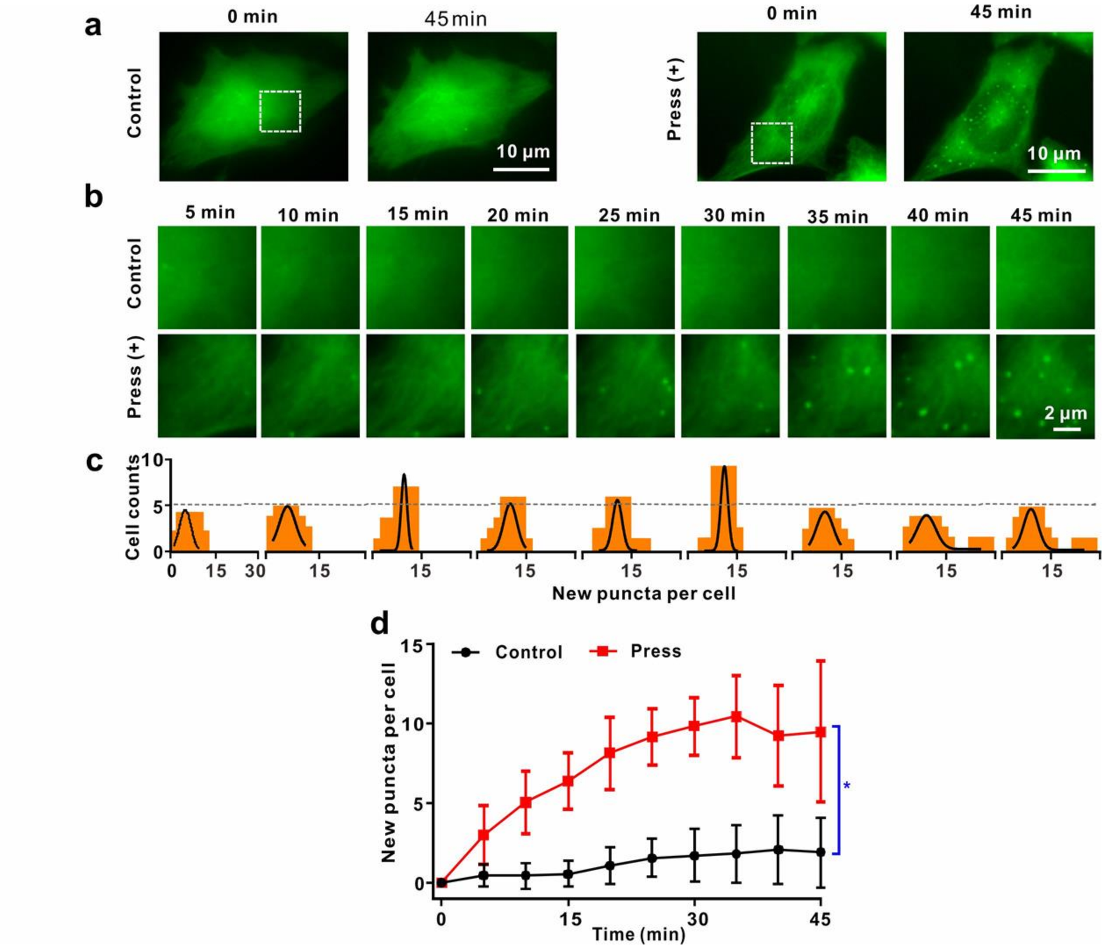

Figure S14. Dynamics of autophagosome formation. a) Fluorescence images of a single HeLa-GFP-LC3 cell before and after 45 min without stimulation (control); and, similarly, following 45 min nanoacupuncture with 5 nN loading force with a spherical tip of 2.5 µm diameter (press (+)), scale bar: 10 µm. b) Inset from a) time course without (control) and with nanoacupuncture (press (+), scale bar: 2 µm. c) Distribution of the numbers of new puncta in cells. d) Summary graph: average number of new puncta per cell vs. time. n = 12; data were presented as mean ±SD. *$p<0.05$; Student's t-test.

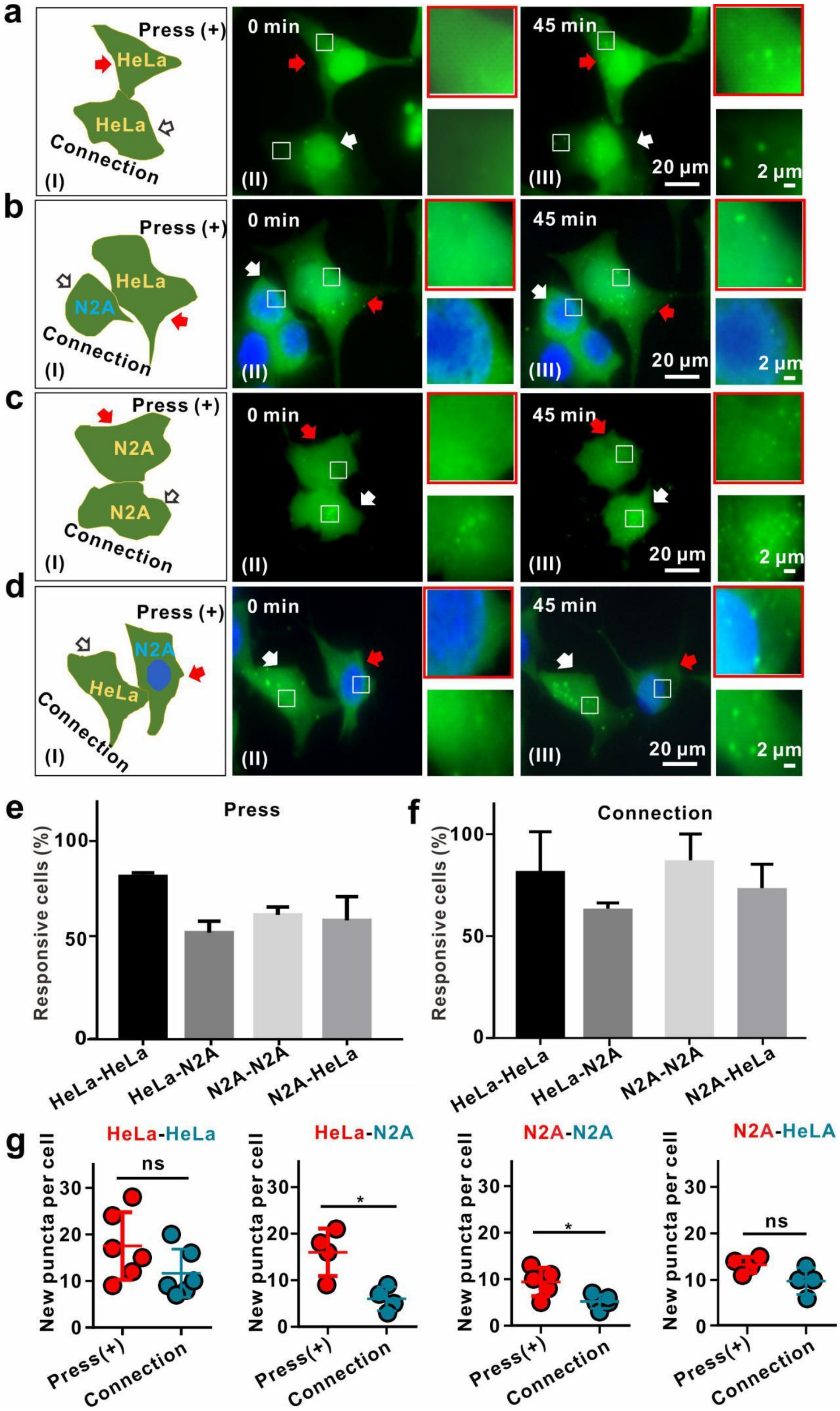

Figure S15. Nanoacupuncture induced autophagy in cell pairs via cell-cell connection. a) HeLa-GFP-LC3 cells (HeLa-HeLa), b) co-cultured HeLa-GFP-LC3 and Hoechst stained N2A-GFP-LC3 cells (N2A-HeLa), c) N2A-GFP-LC3 cells (N2A-N2A), d) co-cultured HeLa-GFP-LC3 and Hoechst stained N2A-GFP-LC3 (HeLa-N2A); (I) Schematic illustration of the cell pairs. Nano acupuncture stimulated cells were defined as press (+) group and indicated by red arrows. Unstimulated contacting cells were defined as connection group and indicated by white arrows. (II) Representative fluorescence images of a single cell pair during

nanoacupuncture at 0 min and (III) 45 min. e) and f) Summary of corresponding percentage of responsive cells and contacting cells with puncta appearance (n = 20, 28, 27 and 25, for HeLa-HeLa, N2A-HeLa, N2A-N2A, and HeLa-N2A, respectively). Data were presented as mean ±SD. Autophagy response could be observed in unstimulated cells connected to cells directly stimulated via nanoacupuncture. g) The average number of new puncta in each responsive cell in pairings of cells. The stimulated (press) group is in red and the connection group is in blue (n = 6, 4, 5 and 4, for HeLa-HeLa, N2A-HeLa, N2A-N2A, and HeLa-N2A, respectively). Data were presented as mean ±SD. Cone-shaped AFM tip diameter: 20 nm; nanoacupuncture time: 45 min; loading force: 5 nN. *$p<0.05$, ns: not significant, Student's t-test.

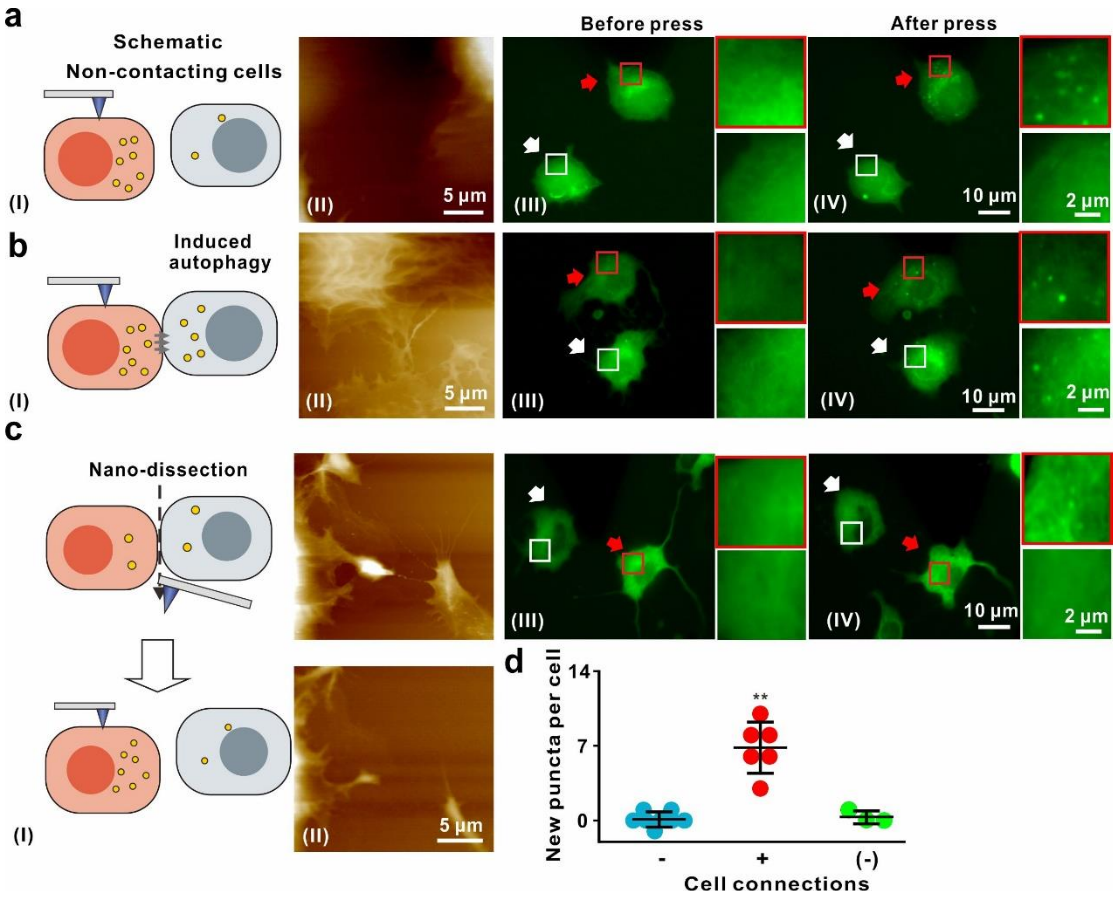

Figure S16. Nanoacupuncture induced autophagosome formation in adjacent contacting N2A-GFP-LC3 cells. Nanoacupuncture induced autophagy in a cell pair without connection a), in a cell pair with direct connection b), and in a cell pair separated by nano-dissection c). (I) The schematic of the cell pair; (II) AFM image (height mode, scale bar 10 µm); (III) the GFP-LC3 fluorescence image of the same cell pair before and (IV) after nanoacupuncture (scale bar 10 µm and 2 µm). The cell undergoing nanoacupuncture are indicated by red arrows and the unstimulated cell are indicated by white arrows. d) Average number of new puncta in each single unstimulated cell. Cell pairs without connections (-, n = 6; data were presented as mean ± SD); cell pairs with connections (+, n = 6; data were presented as mean ± SD); cell pairs with connections resected ((-), n = 3; data were presented as mean ± SD). Loading force: 5 nN; cone-shaped AFM tip diameter: 20 nm; nanoacupuncture time: 45 min. **p<0.01, Student's t-test.

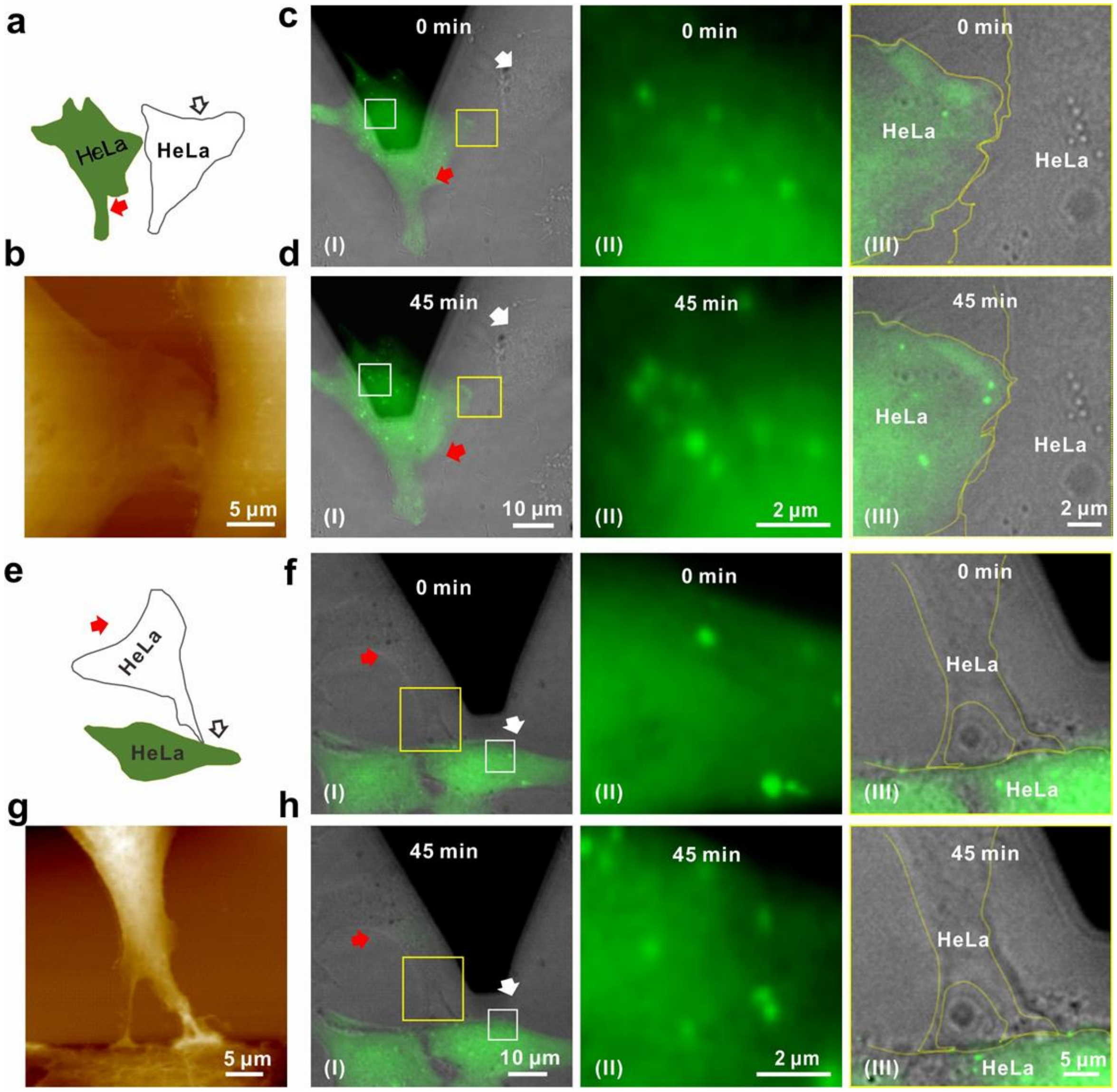

Figure S17. nanoacupuncture induced autophagy in paired cells occurs without autophagosome exchange. a) and e): Schematic illustration of a cell pair of HeLa-GFP-LC3 (green fluorescence) and unlabeled HeLa cells. Cells subjected to nanoacupuncture via the AFM tip are indicated by red arrows. Cells connected to but unstimulated are indicated by white arrows. b) and (g): height mode AFM images of the contacting regions of a single cell pair. c), d), f), and h): merged images of DIC and fluorescence (green) image of AFM tip applying nanoacupuncture. The connection section is enlarged and indicated by a yellow frame. The exchange of autophagosomes-green puncta- was not observed. Cone-shaped AFM tip diameter: 20 nm, nanoacupuncture time: 45 min; loading force: 5 nN.

Table S1. Correlation between the loading force, indentation, and contact area on a HeLa cell membrane using a spherical-shaped AFM tip

No.	SEM image of the AFM tip	Diameter of the spherical-shaped AFM tip (μm)	Loading force (pN)	Indentation depth (nm)	Contact area (μm^2)
1	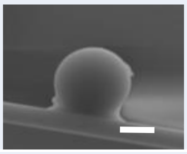	2.5	25	25,	0.2
			50	45	0.4
			100	90	0.7
			300	150	1.2
			500	190	1.5
			1000	215	1.7
			5000	590	4.6
2	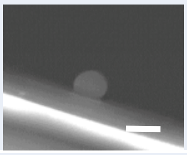	1	5000	610	~1.5
3	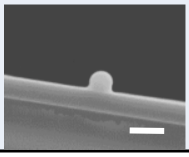	0.6	5000	670	~0.5

Note：1) for the SEM images, Scale bar:1 μm.

2) we define the calculation formula under two different conditions. For the case of indentation depth ≤ probe diameter, contact area = $2\pi Rh$; for the case of indentation depth > probe diameter, contact area = $2\pi R^2$.

Table S2. Heights and spreading areas of HeLa, PC12, and N2A single cells

Cells	Height (µm)										Mean ± SD
HeLa	5.56	6.31	6.21	4.67	5.33	7.1	5.13	5.2	4.66	3.93	5.41 ± 0.2936
PC12	3.67	4.28	4.36	4.85	4.767	4.158	4.566	4.98			4.454 ± 0.1515
N2A	6.81	6.913	9.95	5.22	9.15	6.79	7.32	7.65	7.2	10.59	7.759 ± 0.5193

Cells	Area (μm^2)										Mean ± SD
HeLa	700	927	800	594	384	445	603	341	557	588	593.9 ± 57.38
PC12	455	638	551	680	308	446	302	645	481	291	479.7 ± 46.67
N2A	492	357	361	263	296	408	283	353	295	260	336.8 ± 23.11

3. References

[1] a) P. F. M. T. Arce, G. A. Riera, P. Gorostiza, F. Sanz, *Appl Phys Lett* **2000**, 77, 839; b) K. L. Johnson, K. Kendall, A. D. Roberts, *Proc R Soc Lon Ser-A* **1971**, 324, 301.
